# Use of Telehealth to Support Family Caregivers of Hospice Patients During the COVID-19 Pandemic

**DOI:** 10.1093/geroni/igab046.1799

**Published:** 2021-12-17

**Authors:** George Demiris, Debra Parker Oliver, Karla Washington

**Affiliations:** 1 School of Nursing, University of Pennsylvania, University of Pennsylvania, Pennsylvania, United States; 2 Washington University in St. Louis, St. Louis, Missouri, United States

## Abstract

Family caregivers of hospice patients faced additional challenges in the context of the COVID-19 pandemic where social isolation and loneliness that are often observed among those taking care of a loved one at the end of life, were exacerbated by social distancing rules and workflow changes introduced by hospice agencies. The use of telehealth technologies has the potential to facilitate the delivery of supportive services for family caregivers. We conducted a study examining the use of telehealth for the delivery of a supportive intervention based on problem solving therapy and positive appraisal theory designed specifically to support family caregivers of hospice patients during the COVID-19 pandemic. We recruited 248 caregivers who each participated in three telehealth sessions over a month; caregivers reported higher levels of quality of life and lower levels of anxiety post intervention. Specific recommendations for inclusive telehealth design are discussed based on lessons learned.

